# Treatment of Anthrax

**Published:** 1893-11

**Authors:** 


TREATMENT OF ANTHRAX.—Dr. F. Goilay (*Roumaine Medicale* No. 4, 1893) describes a method of treating anthrax which he has used with success in fourteen cases. After washing the region of the carbuncle with a 4 per cent. solution of boric acid, he opens it with a crucial incision extending a half inch into the surrounding healthy tissue. No anæsthetic is necessary except in very nervous and sensitive persons, where three to four injections of a 1 per cent. solution of cocaine may be used. After opening, it is again washed with the boric acid solution and covered with crystallized boric acid over which is laid a piece of sterilized gauze and a bandage. Under the first dressing, which is allowed to remain on for twenty-four hours, the pains decrease in severity, the temperature falls, the general condition ameliorates and sleep and appetite improve. The next day the dressing is removed, the loose necrotic shreds removed, the carbuncle washed again with the 4 per cent. solution and covered with the crystallized acid. The second dressing remains undisturbed for five to eight days. In twelve of the cases three dressings were sufficient to bring about healing in seven to twenty days. Powdered boric acid is less applicable as it forms a mixture with the pus which hinders recovery. The advantages of this treatment are that it relieves the congestion of the tissues, exposes the pus cavity without opening many blood vessels, is antiseptic and can be carried out without chloroform. It requires but infrequent change of the dressing, produces rapid healing and may be used in any individual or any portion of the body.—*Med. and Surg. Repoiter.*

